# Editorial: Innovative pathological approaches for poorly cohesive gastric cancer

**DOI:** 10.3389/fonc.2026.1972390

**Published:** 2026-09-10

**Authors:** Francesco Vasuri, Luca Saragoni

**Affiliations:** 1 Pathology Unit, Santa Maria delle Croci Hospital, Ravenna, Italy; 2 Department of Medical and Surgical Sciences (DIMEC), Bologna University, Bologna, Italy

**Keywords:** gastric cancer, multidisciplinary approaches, pathology, poorly cohesive carcinoma, target therapy

Poorly cohesive gastric carcinoma (PCGC) represents the most challenging histological subtype of gastric cancer, encompassing a heterogeneous group of carcinomas composed of isolated cells or small, loosely organized clusters, frequently with signet-ring cell morphology, substantially overlapping the diffuse Lauren type; recently PCGC without signet-ring morphology were recognized as well, and grouped under the “not otherwise specified” (NOS) category. This group surely deserves more attention in the future. PCGC infiltrative growth pattern may result in subtle endoscopic and radiological findings, underestimation of tumor extension in superficial biopsies, early peritoneal dissemination, and limited responsiveness to several conventional therapeutic approaches. At the biological level, impaired cell adhesion, epithelial–mesenchymal plasticity, stromal interactions, and molecular heterogeneity contribute to the distinctive behaviour of these tumors (1–3).

Pathologists are therefore required to move beyond descriptive morphology and to integrate histological findings with inherited cancer susceptibility, immunophenotypic and molecular biomarkers, computational analyses, and other multidisciplinary information. This Research Topic was conceived to explore this complementary approach and to highlight how it may improve the detection, characterization, risk assessment, and clinical management of PCGC.

The contribution by Cavallaro et al. addresses one of the most consequential clinical settings associated with diffuse gastric cancer: hereditary diffuse gastric cancer syndrome. The authors describe an Italian family affected by several early-onset, rapidly progressive gastric carcinomas associated with the germline pathogenic CDH1 variant c.1792C>T. Multiple family members developed advanced PCGC at a young age, whereas two asymptomatic female carriers underwent risk-reducing total gastrectomy following genetic counselling, without identification of invasive or intramucosal carcinomas. This apparently discordant combination well illustrates the tension between underdiagnosis and overtreatment: the absence of detectable neoplastic foci in a prophylactic gastrectomy does not mean that surgery was inappropriate, but it rather reflects the incomplete understanding of penetrance, lesion initiation, and progression in individual CDH1-mutated carriers. Recent data suggest that the average lifetime risk of advanced gastric cancer among carriers may be lower than previously estimated, while simultaneously confirming that family history is a major risk modifier (4, 5). The work by Cavallaro et al. therefore supports an individualized approach in which variant interpretation, pedigree analysis, age of onset in affected relatives, patient preferences, and the limitations of endoscopic surveillance are considered as a whole.

Although focused on rectal rather than gastric cancer, the original study by Weng et al. broadens the methodological perspective of the Research Topic by exploring the integration of alternative splicing, mutational data, and immune profiling. Using publicly available transcriptomic and genomic datasets, the authors identified thousands of deregulated alternative splicing events and selected 217 potential tumor-antigen genes through the intersection of upregulated splicing abnormalities and frameshift mutations, e.g. FAM135A, GAR1, and CDIPT. The authors also divided rectal cancers into two immune subtypes, C1 and C2, characterized by different prognostic and immunological profiles. Their analyses suggested potential differences in responsiveness to immune-checkpoint blockade and provided a framework for selecting patients who might benefit from future mRNA vaccine strategies. This paper demonstrated how transcript-level alterations, including abnormal splicing events that are not captured by conventional mutation analysis, may be investigated as sources of neoantigens and as components of immune stratification, a model applicable to gastric cancer as well, albeit the transferability of the proposed classification to gastric cancer remains to be established (3).

The case report by Hui et al. illustrates a different but equally relevant task of modern pathology, i.e. distinguishing metastatic disease from synchronous primary malignancies. The authors describe a patient with a poorly differentiated gastric adenocarcinoma and a large retroperitoneal pleomorphic liposarcoma. The two tumors were resected during the same surgical procedure, and their separate histogenesis was established by detailed morphological and immunohistochemical assessment. The retroperitoneal lesion showed a mesenchymal phenotype, and an immunohistochemical profile supporting its classification as pleomorphic liposarcoma rather than a metastasis. Following gastric surgery and adjuvant treatment, no recurrence was detected during 48 months of follow-up. This unusual association highlights the importance of avoiding the automatic assumption that every additional lesion identified during the staging of a patient with cancer represents metastatic disease, since it may lead to incorrect staging, inappropriate therapeutic decisions, or denial of potentially curative surgery.

Finally, the review by Muratore et al. provides the disease-specific framework that most directly reflects the scope of this Research Topic, revising the pathological definition of PGCG subgroups and connecting histological classification with the expanding biomarker landscape of gastric cancer. Particular attention is given to adequate biopsy sampling and pre-analytical control, limited diagnostic material, and marked intratumoral heterogeneity. The authors then critically discuss HER2, PD-L1, CLDN18.2, FGFR2 and FGFR2b, and MSI/dMMR, together with emerging targets and therapeutic platforms such as TROP2-directed agents, antibody-drug conjugates, and bispecific antibodies. A central message of this contribution is that therapeutic innovation in gastric cancer has largely been developed through biomarker-selected trials enrolling histologically unselected populations: consequently, the specific clinical value of each single marker in PCGC remains incompletely defined.

Taken together, the four contributions collected in this Research Topic illustrate different but complementary levels at which pathology can influence the management of PCGC, reflecting at the same time the heterogeneous and broader evolution of oncological pathology. Innovation should not be identified exclusively with technological complexity: a refined histological definition, rational sampling and pre-analytical control, recognition of a hereditary syndrome, correct classification of an unexpected second malignancy, and a multimodal computational model can all be innovative when they resolve a clinically relevant uncertainty. Future research should combine standardised pathological diagnosis with tissue biomarker assessment, clinically annotated collaborative datasets, digital pathology, spatial profiling, artificial intelligence, and multi-omics. Bridging these diagnostic and therapeutic gaps offers the most credible route towards a more precise and biologically informed management of patients with PCGC.
